# Supplementary material for: Prediction model for periodontitis stage based on the salivary microbiome
Source: mSystems. 2026 Mar 11;11(4):e01103-25. doi: 10.1128/msystems.01103-25 (PMC13098277; doi:10.1128/msystems.01103-25)
Supplement: Figure S1 — Absolute abundance of salivary bacterial taxa in the different periodontal statuses at the species level. [file msystems.01103-25-s0001.pdf]

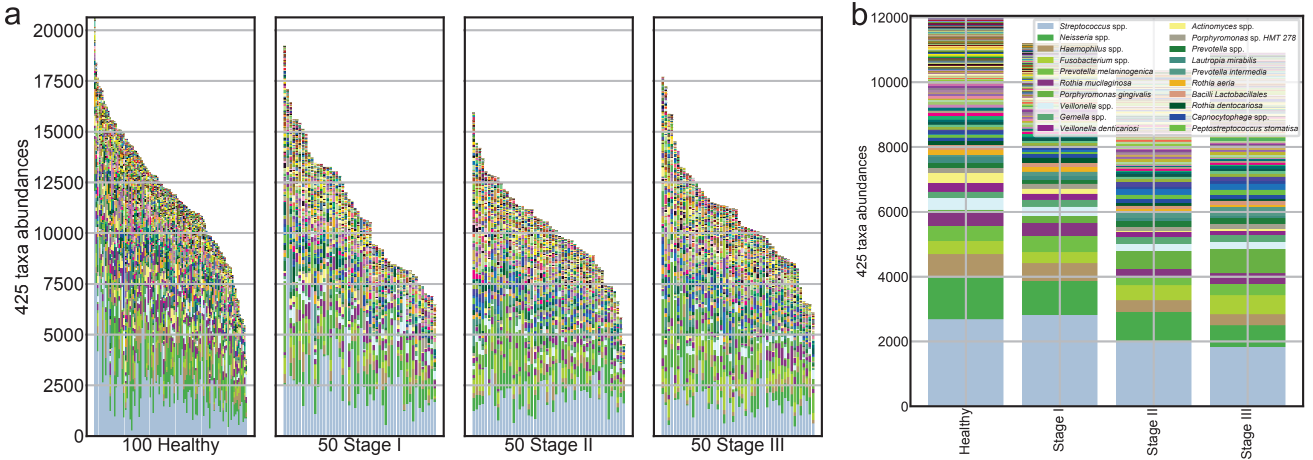

Supplementary Figure 1. Absolute abundance of salivary bacterial taxa in the different periodontal statuses at the species level

Stacked bar plot of the absolute abundance of bacterial species for all samples (a) and the mean absolute abundance of bacterial species in the healthy, stage I, stage II, and stage III groups (b
