# Supplementary material for: Prediction model for periodontitis stage based on the salivary microbiome
Source: mSystems. 2026 Mar 11;11(4):e01103-25. doi: 10.1128/msystems.01103-25 (PMC13098277; doi:10.1128/msystems.01103-25)
Supplement: Table S1 — Beta-diversity pairwise comparisons on the periodontitis stages. [file msystems.01103-25-s0002.pdf]

| Group 1 | Group 2 | p-value |
|---|---|---|
| Healthy | Stage I | 0.001 |
| Healthy | Stage II | 0.001 |
| Healthy | Stage III | 0.001 |
| Stage I | Stage II | 0.001 |
| Stage I | Stage III | 0.001 |
| Stage II | Stage III | 0.737 |

**Supplementary Table 1. Beta-diversity pairwise comparisons on the periodontitis stages**

Statistically significant (p-value) was determined by the PERMANOVA test.
