# Supplementary material for: Prediction model for periodontitis stage based on the salivary microbiome
Source: mSystems. 2026 Mar 11;11(4):e01103-25. doi: 10.1128/msystems.01103-25 (PMC13098277; doi:10.1128/msystems.01103-25)
Supplement: Table S2 — Feature importance of taxa in the classification of different periodontitis stages. [file msystems.01103-25-s0003.pdf]

Condition	Healthy vs. Stage I vs. Stage II vs. Stage III		Healthy vs. Stage I		Healthy vs. Stage I vs. Stage II/III		Healthy vs. Stage I/II/III	
Rank	Taxa	Importance	Taxa	Importance	Taxa	Importance	Taxa	Importance
1	*Actinomyces* spp.	0.306	*Actinomyces* spp.	0.252	*Actinomyces* spp.	0.417	*Actinomyces* spp.	0.530
2	*Corynebacterium durum*	0.084	*Actinomyces graevenitzii*	0.083	*Corynebacterium durum*	0.105	*Porphyromonas gingivalis*	0.082
3	*Treponema* spp.	0.063	*Porphyromonas gingivalis*	0.072	*Porphyromonas gingivalis*	0.075	*Actinomyces graevenitzii*	0.052
4	*Actinomyces graevenitzii*	0.058	*Treponema* spp.	0.070	*Actinomyces graevenitzii*	0.063	*Corynebacterium durum*	0.040
5	*Porphyromonas gingivalis*	0.056	*Peptostreptococcaceae [XI][G-9] brachy*	0.060	*Treponema putidum*	0.041	*Treponema* spp.	0.039
6	*Treponema putidum*	0.044	*Corynebacterium durum*	0.058	*Treponema* spp.	0.037	*Treponema putidum*	0.039
7	*Porphyromonas* sp. *HMT 285*	0.044	*Porphyromonas* sp. *HMT 285*	0.051	*Peptostreptococcaceae [XI][G-9] brachy*	0.028	*Campylobacter showae*	0.029
8	*Peptostreptococcaceae [XI][G-9] brachy*	0.040	*Lachnospiraceae [G-8] bacterium HMT 500*	0.047	*Porphyromonas* sp. *HMT 285*	0.028	*Peptostreptococcaceae [XI][G-9] brachy*	0.027
9	*Campylobacter showae*	0.032	*Campylobacter showae*	0.041	*Campylobacter showae*	0.028	*Porphyromonas* sp. *HMT 285*	0.026
10	*Filifactor alocis*	0.029	*Peptostreptococcaceae [XI][G-6] nodatum*	0.037	*Prevotella intermedia*	0.025	*Prevotella intermedia*	0.024
11	*Peptostreptococcaceae [XI][G-6] nodatum*	0.028	*Treponema putidum*	0.033	*Lachnospiraceae [G-8] bacterium HMT 500*	0.019	*Filifactor alocis*	0.017
12	*Prevotella intermedia*	0.027	*Peptostreptococcaceae [XI][G-5] saphenum*	0.031	*Treponema* sp. *HMT 260*	0.018	*Peptostreptococcaceae [XI][G-5] saphenum*	0.016
13	*Prevotella* sp. *HMT 526*	0.026	*Prevotella intermedia*	0.030	*Mycoplasma faucium*	0.017	*Lachnospiraceae [G-8] bacterium HMT 500*	0.015
14	*Treponema* sp. *HMT 260*	0.025	*Mycoplasma faucium*	0.025	*Filifactor alocis*	0.017	*Mycoplasma faucium*	0.013
15	*Lachnospiraceae [G-8] bacterium HMT 500*	0.025	*Filifactor alocis*	0.024	*Tannerella forsythia*	0.015	*Peptostreptococcaceae [XI][G-6] nodatum*	0.011
16	*Tannerella forsythia*	0.025	*Tannerella forsythia*	0.024	*Peptostreptococcaceae [XI][G-5] saphenum*	0.014	*Fretibacterium* spp.	0.011
17	*Mycoplasma faucium*	0.024	*Prevotella* sp. *HMT 526*	0.022	*Prevotella* sp. *HMT 526*	0.014	*Prevotella* sp. *HMT 526*	0.009
18	*Peptostreptococcaceae [XI][G-5] saphenum*	0.023	*Treponema* sp. *HMT 260*	0.019	*Prevotella* sp. *HMT 304*	0.013	*Tannerella forsythia*	0.007
19	*Prevotella* sp. *HMT 304*	0.022	*Fretibacterium* spp.	0.012	*Peptostreptococcaceae [XI][G-6] nodatum*	0.013	*Treponema* sp. *HMT 260*	0.007
20	*Fretibacterium* spp.	0.021	*Prevotella* sp. *HMT 304*	0.009	*Fretibacterium* spp.	0.012	*Prevotella* sp. *HMT 304*	0.005

Supplementary Table 2. Feature importance of taxa in the classification of different periodontitis stages

Taxa are ranked in descending order of importance, from most important to least important.
